# 

**Published:** 1924-07

**Authors:** 


					JULY, 1924.
VoL XLI- No. 153.
THE
BRISTOL
medico-chirurgical
NAL
THE BRISTOL MEDICO-CHIRURGIC,
Editor: P. WATSON-WILLI A'
WITH WHOM ARE ASSOCIATED
JAMES SWAIN, C.B., C.B.E., M.S., M
J. LACY FIRTH, M.S., M.D., CAREY COOMBS, M.D
J. A. NIXON, C.M.G., M.D., Assistant Editor.
Editorial Secretary: A. L. FLEMMING, M.B., B.Ch.
BRISTOL: J. W. ARROWSMITH LTD., n QUAY STREET.
London : J. w. Arrowsmith (London) Ltd., 6 Upper Bedford Place,
Russell Square.
Price Three Shillings net.

				

